# Determinants of physical activity: A path model based on an ecological model of active living

**DOI:** 10.1371/journal.pone.0220314

**Published:** 2019-07-26

**Authors:** Hsin-Yen Yen, Ching Li

**Affiliations:** 1 School of Gerontology Health Management, College of Nursing, Taipei Medical University, Taipei, Taiwan; 2 Graduate Institute of Sport, Leisure, and Hospitality Management, National Taiwan Normal University, Taipei, Taiwan; Karlsruher Institut fur Technologie, GERMANY

## Abstract

Maintaining physical activity is necessary to promote health in our daily lives. The Ecological Model of Active Living was proposed to examine whether individuals participate in active living. The purpose of this study was to understand the determinants of physical activity and create a predictive path model of the intrapersonal, perceived environment, behavior settings, and policy environment domains. Data were obtained from open government data and questionnaires, including the International Physical Activity Questionnaire, Health Belief Model Inventory, Physical Activity Neighborhood Environment Survey, and Accessibility of Open Spaces. Participants comprised 1085 healthy adults who completed a self-reported internet survey. An analysis of the intrapersonal domain revealed that the variables of female, an older age, and a low educational level, and individuals with obesity or cardiometabolic diseases presented lower odds ratios for active living. We found significant statistical support for our path model (The Ecological Model of Active Living), with a predictive power of 23.1%. The predictive path model is a good approach to quantitatively measure the impacts of various determinants on active living that suggests further lines of research in approaches for modeling relationships.

## 1. Introduction

The World Health Organization identifies physical inactivity as the fourth leading cause of death. The definition of physical activity (PA) is any kind of bodily movement that requires energy expenditure [1]. Physical inactivity leads to low and insufficient energy expenditures in daily living, which contributes to obesity, type 2 diabetes, cardiovascular diseases, cancers, and other chronic diseases [2]. Although engaging in PA to gain sufficient energy expenditure is known to benefit one’s health, over 20% of the population worldwide follows an inactive lifestyle [3]. Public health practitioners have suggested using environmental and psychological perspectives to promote PA.

A socio-ecological model is commonly used to explain the determinants of healthy behaviors. Changes in policy, the environment, and individuals affect and support healthy behaviors [4]. Sallis [5] proposed an Ecological Model of Active Living. This model constructs a framework and examines factors associated with PA, in order to achieve the main goal of active living. Active living focuses on transport and recreational domains of PA which are modifiable by policies, the environment, and individuals’ efforts. The Ecological Model of Active Living is influenced by four domains: (a) intrapersonal domain, including demographic, biological, and psychological effects; (b) perceived environment domain, including accessibility, convenience, attractiveness, and comfort; (c) behavior setting domain, including access and characteristics of the neighborhood, workplace, recreation, and home environments; and (d) policy environment domain, including policies on physical education, facility access, transportation, land use, and park management. The following paragraphs describe details of the four domains.

### Intrapersonal domain

The intrapersonal domain contains three elements: individuals’ demographic and biological characteristics, and psychological effects. Personal attitudes and beliefs predict whether individuals engage in healthy behaviors. According to knowledge, socio-psychological, and structural elements, individuals have their own personal attitudes, health beliefs, and healthy behaviors [6]. The Health Belief Model in Physical Activity (HBMPA) is a common approach to explain PA engagement from a psychological perspective [7]. Concepts of the HBMPA include perceived susceptibility to serious health problems, expected benefits of exercise, barriers to exercise, support of significant others for exercise, and cues to action. Concepts from the HBMPA are widely used to explain individuals’ psychological factors associated with PA. The concepts of HBMPA impact individuals’ decisions leading to different outcomes of PA engagement. From previous studies, social support, cues to action, and benefits of exercise are positively correlated with the level of PA. Declines in PA are aggravated by barriers to exercise [8]. People with higher HBMPA scores have a higher possibility of achieving minimal PA recommendations. In addition, demographic factors such as gender, age, disposable income, and an individual’s health status also impact PA participation [9].

### Perceived environment domain

The living environment is the foundation of residents’ daily lives, PA, and social interactions [10]. The walkability of the neighborhood environment plays an important role in active living. A supportive environment modifies physical facilities and transportation systems to promote walking and cycling behaviors [11]. A high-quality neighborhood provides accessible infrastructure, safety, social supportiveness, low traffic volumes, public transportation, and favored esthetics in the streets [12]. A higher level of walkability gives pedestrians greater positive support and fewer negative influences from the environment, which studies have shown increases their willingness to engage in walking behaviors and PA [10]. Furthermore, walkability is positively correlated with adults’ PA engagement, especially in the transportation and recreational domains [13].

### Behavior setting domain

Public open space (POS) systems should be taken into consideration. POS systems provide spaces for recreation and leisure activities and are also important destinations for residents to interact with each other, such as sport facilities, parks, and community centers. Transportation systems that support commutes by bike or on foot are also POS systems. These characteristics of POS systems encourage residents to participate in PA [14]. The categories, number, distances, areas, functions, and quality of POS systems impact the use of public facilities [15]. A high density and accessibility of public infrastructure can promote the effectiveness of residents’ PA [16].

### Policy environment domain

Creating a healthy environment is the ultimate mission of government. Redesigning walkable and health-supportive environments promotes active lifestyles by residents [17]. Policy decisions have huge impacts that can bring about positive or negative outcomes. Walkability indices (WIs) were developed to evaluate outcomes of policy decisions, such as street connectivity, land use mixes, residential densities, socioeconomic status, and safety [12,18]. PA was found to have positive correlations with WIs because residents living in higher-WI environments have greater opportunities for and frequencies of PA [19,20]. WIs are an indicator of policy performances that determines residents’ active living.

To sum up, PA is a key strategy for promoting health and preventing disease. It is important to increase PA and build active lifestyles [21] from psychological, environmental, and political efforts. The Ecological Model of Active Living is a popular theory which is used to explore effects of the intrapersonal, perceived environment, behavior settings, and policy environment domains of PA. However, previous evidence-based studies seldom discussed simultaneous interactions of psychological and environmental factors [22]. To our best knowledge, no studies have tried to explore relationships among psychological, environmental, and political factors by a path analysis or create a path model of the Ecological Model of Active Living to predict individuals’ PA behaviors.

Therefore, the main purpose of this study was to create a predictive path model that refers to domains from the Ecological Model of Active Living. The path model is comprised of the intrapersonal, perceived environment, behavior setting, and policy environment domains, in order to determine relationships between the correlates and latent variables of PA. Impacts of the intrapersonal domain (demographic and biological characteristics) on active living are also considered. The research questions were as follows:

1. Can elements of the intrapersonal domain (i.e., demographics, including gender, age, and income; and biological, such as the body-mass index (BMI) and cardiometabolic diseases) predict active living?
2. Can the path model for the Ecological Model of Active Living (encompassing the intrapersonal (psychological), perceived environment, behavior setting, policy environment, and active living domains) be accepted?

## 2. Materials and methods

### 2.1 Participants and sampling

Participants were healthy individuals without physical or mental disabilities in Taiwan. Using convenience sampling, we posted an internet survey on social media and other websites from November 2016 to June 2017. Because of possible biases in internet surveys, we only selected participants between 18 and 60 years, in order to better model the distribution of the adult population. Moreover, according to Hill (1998), if the population exceeds 100,000 and the margin of error is 3%, the number of participants in samples should be greater than 1067 to be representative of the population in an internet survey [23]. In total, 1331 participants responded, with a valid response rate of 81.52%. Ethical approval for this study was obtained from the National Taiwan Normal University Research Ethics Committee (NTNU-REC 201605HM025). All participants were required to sign an informed consent form before they were allowed to fill out the anonymous questionnaires.

### 2.2 Measurements

#### 2.2.1 Questionnaires

The personal questionnaire had five parts. The first concerned personal health information. Participants’ background information included gender, age, residence, personal income, educational level, height, and weight. Participants were also asked whether they had chronic diseases, including hypertension, diabetes, and hyperlipidemia.

The International Physical Activity Questionnaire Long Form (IPAQ) is used to measure participants’ PA over the preceding 7 days. The IPAQ assesses the intensity, duration, and frequency of four domains including recreation, domestic and gardening activities, work, and transport. The current study used only two domains which could be influenced by factors of the Ecological Model of Active Living. According to the scoring protocol and participants’ self-reported transport and recreational domains of PA, metabolic equivalents of task (METs) were calculated as a participant’s weekly energy expenditure. The IPAQ Taiwanese version was shown to have good content validity and test-retest reliability [24]. This study was authorized by the Ministry of Health and Welfare, Taiwan. Cronbach’s α of the IPAQ was 0.918 in this study.

Hayslip et al. [25] developed an inventory for the HBMPA. They used the Health Belief Model to explain personal exercise and PA as the main health behaviors. The response options range from 1 to 5 on a Likert scale (strongly disagree, disagree, neutral, agree, and strongly agree). The inventory was translated into a Chinese version with good validity [26]. The Chinese version also has the same factors as the original inventory, including perceived susceptibility to serious health problems, expected benefits of exercise, barriers to exercise, support of significant others for exercise, and cues to action. The inventory of the HBMPA presented good reliability with Cronbach’s α of HBMPA was 0.917 in this study.

The Physical Activity Neighborhood Environment Survey (PANES) is used to measure self-reported walkability in the perceived environment domain. The PANES was developed by Sallis and Saelens [27]. The items in the survey concern attributes of the neighborhood within 10~15 minutes of home by walking. The response options are mainly scored on a 4-point Likert scale (strongly disagree, disagree, agree, and strongly agree). The PANES was tested in 11 countries and showed test-retest reliability and criterion validity with the IPAQ [28]. According to the scoring protocol (How to score PANES; [29], the sum of items presents participants’ perceived walkability of open spaces. Cronbach’s α of the PANES Chinese version was 0.715 in this study.

The questionnaire on the accessibility of open spaces for PA was developed for the behavior setting domain, and refers to part 2 of the Neighborhood Environment Walkability Scale-Traditional Chinese long version (NEWS-C). Items in the NEWS-C are related to various facilities and services in the neighborhood (i.e., supermarkets, bookstores, pharmacies, libraries, and post offices) [29]. All 11 items in this study focused on open spaces for PA, including parks, squares, pathways, public sports centers, private recreational facilities, swimming pools, and community centers. The frequency of use (less than once a month, two or three times a month, once or twice a week, three to four times a week, and more than five times a week), the difficulty of transport (by car, motorcycle, public transportation, bicycle, and on foot), and the time of transport from home (>30, 21~30, 11~20, 6~10, and <5 minutes) to the open spaces for PA were each measured on a 5-point Likert scale. Respective scores for each open space were added up to calculate the accessibility, where a higher score indicates better accessibility of spaces for PA. The amounts of transport and recreational PA were used as criteria for tests of the criterion-related validity. The correlation coefficient between PA and accessibility was 0.99 (*p*<0.05). Cronbach’s α of the accessibility questionnaire was 0.762.

#### 2.2.2 Open government data (OGD)

OGD were used for measurements in the policy environment domain which were separate from the internet survey. The goals of OGD are to integrate public departments and improve public services [30]. OGD are also a good indicator of the efficacy of policies from planning to execution [31]. Based on previous studies [12,18,32], several indicators of the policy environment of PA were selected, including street connectivity (i.e., the coverage rate of sidewalks and stations), land use mix (i.e., the coverage rate of parks, squares, and sports facilities), residential density (i.e., the ratio of the population to the area of a residential area), socioeconomic status (family incomes), and safety (crime rates). OGD in 2015 were retrieved from local government bodies (National Statistics, Taiwan; [33]) for indicators of living areas. The indices were standardized to Z-scores and summed to yield a WI in order to examine the efficacy of policies in the respective administrative regions. According to each participant’s current address, they were assigned a WI number as an indication of the performance of the policy environment domain.

### 2.3 Data analysis

After data were collected, incomplete responses or missing data, outliers, and participants not aged 18~60 years were excluded (*n* = 246). Based on the sum of transport and recreational PA, participants were divided into two patterns of living, inactive living (light: PA <600 MET-min/week) and active living (moderate to vigorous: ≥600 MET-min/week) [34]. The mean of subscales of five factors in the HBMPA inventory was calculated. SPSS statistical software was used for the descriptive analyses, independent *t*-test, and logistic regression. Scores of transport and recreational PA, subscales of the HBMPA, PANES, accessibility, and WI were transformed into Z-scores in the path analysis. Smart-PLS 3.0 software was used to create a path model.

## 3. Results

### 3.1 Characteristics of participants

Demographic characteristics of 1085 participants stratified into active living and inactive living groups are shown in Table 1. Table 2 also illustrates results of the *t*-test between the two groups. There were significant differences in age, benefits of exercise, barriers to exercise, perceived walkability, accessibility, WI, and PA between the active and inactive living groups.

**Table 1:** Characteristics of participants.

Background		Total		Inactive living		Active living	
		*n*	%	*n*	%	*n*	%
**Gender**	Male	534	49.22%	139	26.03%	395	73.97%
	Female	551	50.78%	198	35.93%	353	64.07%
**Educational level**	High school	135	12.44%	46	34.07%	89	65.93%
	College	666	61.38%	217	32.58%	449	67.42%
	Graduate school	284	26.18%	74	26.06%	210	73.94%
**Income (NT$)**	<500,000	469	43.23%	141	30.06%	328	69.94%
	500,001~750,000	250	23.04%	87	34.80%	163	65.20%
	750,001~1,000,000	202	18.62%	54	26.73%	148	73.27%
	>1,000,001	164	15.12%	55	33.54%	109	66.46%
**Employment status**	Student	345	31.80%	91	26.38%	254	73.62%
	Employed	653	60.18%	222	34.00%	431	66.00%
	Unemployed^1^	87	8.02%	24	27.59%	63	72.41%
**Body-mass index (kg/m**^**2**^**)**	Normal (<24)	758	69.86%	230	30.34%	528	69.66%
	Overweight (24~27)	186	17.14%	54	29.03%	132	70.97%
	Obese (>27)	132	12.17%	51	38.64%	81	61.36%
**Hypertension**	Yes	96	8.89%	23	23.81%	73	76.19%
	No	989	91.11%	193	19.51%	796	80.49%
**Diabetes**	Yes	30	2.75%	9	30.77%	21	69.23%
	No	1055	97.25%	207	19.59%	848	80.41%
**Hyperlipidemia**	Yes	162	14.93%	43	26.43%	119	73.57%
	No	923	85.07%	174	18.86%	749	81.14%
**Cardiometabolic disease**^2^	Yes	276	25.44%	95	34.42%	181	65.58%
	No	809	74.56%	242	29.91%	567	70.09%
**Total**		**1085**	**100.00%**	**337**	31.06%	**748**	**68.94%**

^1^ Unemployed included those with a part-time job, a stay-at-home parent, retiree, or a home office without a fixed salary.

^2^ Cardiometabolic diseases represent individuals with hypertension, diabetes, or hyperlipidemia.

The exchange rate in May 2019 was US$1≈New Taiwan (NT)$30.50.

**Table 2:** Descriptive results of participants.

Variable	Total		Inactive living		Active living		MD^1^	*t*	
	Mean	SD	Mean	SD	Mean	SD			
Age (years)	28.90	8.12	29.96	9.27	28.43	7.51	1.53	2.67	**
**Intrapersonal domain (Health beliefs in PA)**									
Susceptibility to health problems	3.18	0.90	3.15	0.85	3.20	0.92	-0.05	-0.92	
Benefits of exercise	4.03	0.65	3.81	0.66	4.14	0.62	-0.33	-7.89	***
Barriers to exercise	2.35	0.68	2.61	0.56	2.23	0.69	0.37	9.44	***
Cues to action	3.29	0.80	3.25	0.70	3.31	0.83	-0.07	-1.36	
Significant others’ support	3.27	0.86	3.22	0.78	3.30	0.90	-0.09	-1.61	
**Perceived environment, Behavior setting, Policy environment domains**									
Perceived walkability	44.65	6.39	43.17	6.06	45.32	6.43	-2.15	-5.18	***
Accessibility	37.31	37.87	23.15	28.94	43.69	39.67	-20.53	-9.59	***
Walkability Index	1.14	3.13	0.60	3.03	1.39	3.15	-0.79	-3.92	***
**Active living (PA)**									
Transport PA (MET-min/week)	302.33	488.47	73.04	129.08	405.63	551.67	-332.59	-15.57	***
Recreational PA (MET-min/week)	1382.64	1525.14	134.19	174.52	1945.11	1530.29	-1810.92	-31.91	***
Total (MET-min/week)	1684.97	1674.26	207.24	202.03	2350.74	1618.77	-2143.51	-35.60	***

^1^ SD, standard deviation; MD, mean difference; PA, physical activity; MET, metabolic equivalent of task.

* *p*<0.05,

** *p*<0.01,

*** *p*<0.001.

### 3.2 Intrapersonal domains predict active living

Results of the logistic regression for the intrapersonal domain are shown Table 3. Dependent variables of gender, age, educational level, and individuals with obesity or cardiometabolic diseases influenced whether participants engaged in active living. Results of the odds ratio (OR) for gender indicated that a woman was 0.58-times as likely as a man to participate in active living. Furthermore, there was an interaction between gender and age for active living. Males and females reflect different directions of the ORs for active living. With an increase of 1 year in age, men showed an increase of 1.01-fold for active living; however, women showed a decrease of 0.02-fold for active living. The OR for educational level indicated that participants with a graduate school certification were 2.08-times more likely than participants with a high school certification to participate in active living. Furthermore, an individual with obesity or a cardiometabolic disease was less likely to participate in active living than was a healthy individual. The OR for income was not significantly associated with active living. The *R*^2^ of the logistic regression model was 0.05.

**Table 3:** Intrapersonal domains predicting active living by the logistic regression.

Intrapersonal domain		B	SE	Wald	OR		95% CI	
**Gender**	Male	(reference)						
	Female	-0.54	0.14	14.93	0.58	***	0.44	0.77
**Age**		-0.03	0.01	11.48	0.97	***	0.95	0.99
	Male × Age	0.01	0.00	8.40	1.01	**	1.00	1.02
	Female × Age	-0.02	0.00	19.92	0.98	***	0.97	0.99
**Educational Level**	High school	(reference)						
	College	0.35	0.22	2.61	1.42		0.93	2.19
	Graduate school	0.73	0.26	8.15	2.08	***	1.26	3.44
**Income**^1^	<500,000	(reference)						
	500,001~750,000	-0.06	0.18	0.11	0.94		0.66	1.35
	750,001~1,000,000	0.27	0.21	1.67	1.31		0.87	1.99
	>1,000,001	-0.08	0.24	0.11	0.93		0.58	1.48
**Body-mass index**	Normal	(reference)						
	Overweight	0.03	0.19	0.03	1.04		0.72	1.49
	Obese	-0.39	0.20	3.55	0.68	*	0.46	1.02
**Chronic diseases**	None	(reference)						
	Cardiometabolic	-0.41	0.19	4.77	0.66	*	0.46	0.96
	Constant	2.22	0.36	38.32	0.00			

Nagelkerke’s *R*^2^ = 0.051;

* *p*<0.05,

** *p*<0.01,

*** *p*<0.001.’ SE, standard error; OR, odds ratio; CI, confidence interval.

^1^ The exchange rate in May 2019 was US$1.00≈New Taiwan (NT)$30.50.

### 3.3 The path model for the ecological model of active living

**Fig 1:**
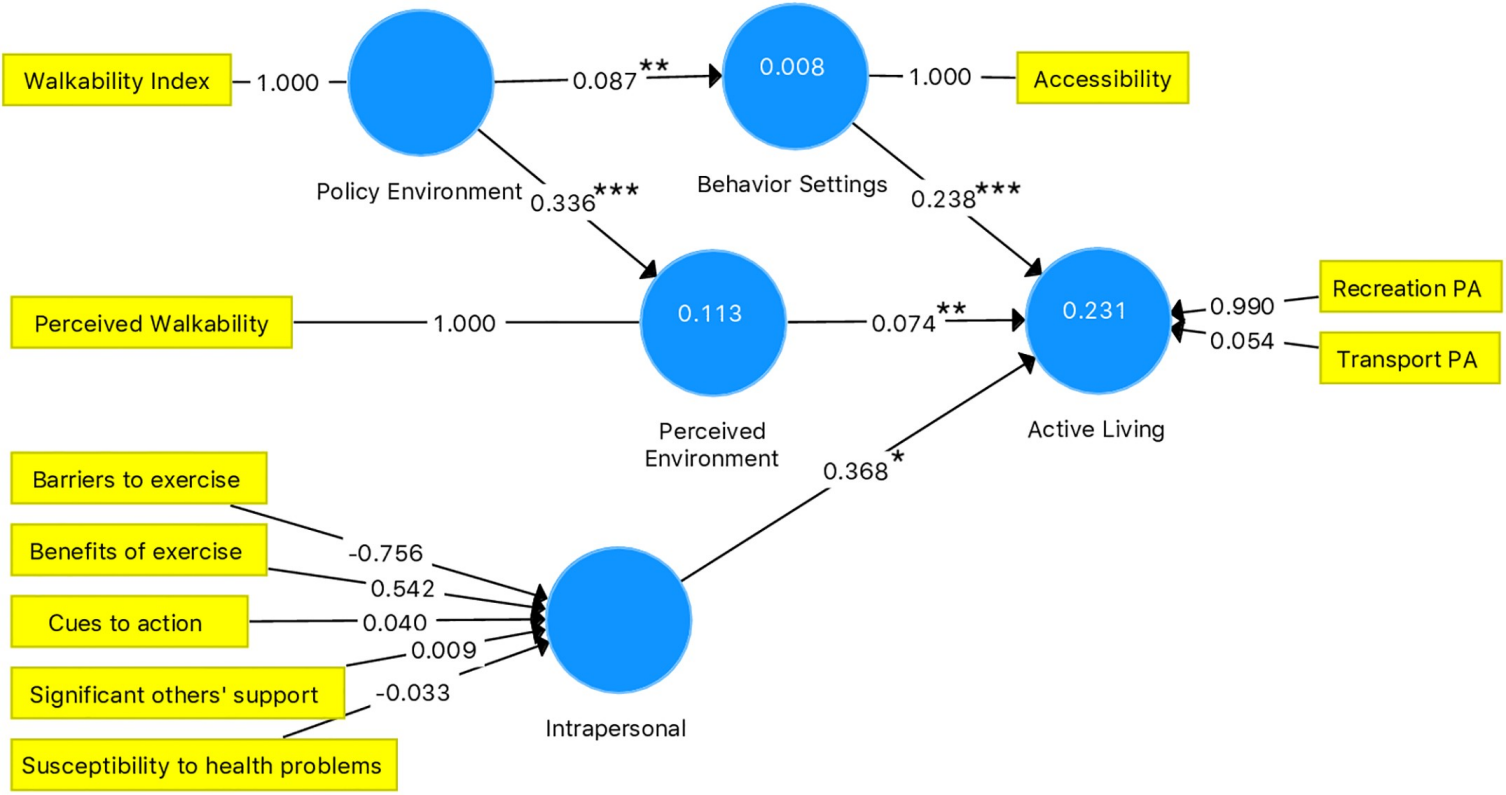
A path model of active living.

Results of the path model of our Ecological Model of Active Living are illustrated in Fig 1. In the latent variable of the intrapersonal domain, values of the indicators’ variance inflation factor (VIF) ranged 1.09~2.28. In the latent variable of active living, VIF values of recreational PA and transport PA were 1.03 and 1.03 (VIF <10). This indicates that collinearity was not high between the predictive constructs in this model.

The path coefficient between the policy environment and perceived environment domains was 0.336 (*t* = 13.67, *p*<0.001), and the *R*^2^ value was 0.113, while that between policy environment and behavior setting domains was 0.087 (*t* = 3.27, *p*<0.01), with an *R*^2^ value of 0.008. The coefficient of determination was considered a weak predictor of the perceived environment and behavior setting domains. The path coefficient between the intrapersonal and active living domains was 0.368 (*t* = 2.42, *p*<0.05), that between the perceived environment and active living domains was 0.074 (*t* = 2.688, *p*<0.01), and that between the behavior setting and active living domains was 0.238 (*t* = 7.28, *p*<0.001), with an *R*^2^ value of 0.231, which indicated that it was a moderate predictor of active living. All path coefficients were significant in this path model. The Normed Fit Index (NFI) was 0.96 (>0.90), and the standardized root mean square residual (SRMR) value was 0.041 (<0.08), so this path model of active living was accepted.

## 4. Discussion

This study investigated the environmental determinants of PA according to the Ecological Model of Active Living. In order to answer the main research question, this study created a predictive path model based on the Ecological Model of Active Living. This path model of active living was statistically accepted with a predictive power of PA of 23.1%. Public health practitioners can encourage individuals’ PA from perspectives of the intrapersonal, perceived environment, behavior setting, and policy environment domains. Instead of an ecological model, Lee [35] applied the Planning Behavior Theory to create a structural equation model of PA whose results were similar to those of our study.

This empirical finding provides an accepted path model for the Ecological Model of Active Living with predictive power to support the research question. Indeed, the Ecological Model can predict physically active living. According to the path model, the policy environment domain influenced the behavior setting and perceived environment domains. The WI, compiled from open data, concerns development of local services and facilities in the community. It is also related to the utility of neighborhood settings [13]. The WI directly influences residents’ feeling of walkability in their neighborhood environment due to such factors as infrastructure, streets, safety, social capital, transportation, and esthetics [36]. Objective and subjective measures of walkability showed a positive correlation [18]. The high accessibility and density of recreational facilities can increase the utility and amount of PA [37]. Based on results of the current study, policymakers should work on developing sports facilities, parks, urban planning, transportation, and safety policies to build a highly walkable environment, in order to increase residents’ PA and maintain active living.

Furthermore, the intrapersonal and perceived environment domains were both important determinants of PA. The HBMPA was applied as latent variables of the intrapersonal domain in the path model, and five factors impacted active living from a psychological perspective. High perceived benefits of exercise, low perceived barriers to exercise, sufficient cues to action, and significant others’ support can increase PA. Previous studies also demonstrated that factors in the HBMPA had significant associations with sports participation, PA, and energy expenditure [8,38]. Based on results of the current study, from a psychological perspective, public health practitioners should provide physical education courses to help all age groups understand the benefits of PA and overcome barriers to PA. Providing sufficient cues to healthy actions can motivate individuals’ PA. Social support through long-term positive relationships of family, friends, and neighbors can also easily change a person’s intention to engage in PA [39].

This study also found that several elements of the intrapersonal domain could predict engagement in active living for addressing the other research question. Results of the demographic variables also suggested that men had higher odds of participating in active living than women. With age, the odds of participating in active living decrease. Several studies demonstrated the influence of gender and age on PA. A male adult spends more time and energy expenditure on PA at a greater frequency than does a female adult, while older adults have a lower probability of achieving goals of PA recommendations than do younger adults [40,41]. This study also found an interaction between age and gender for active living. The aging process affects males’ and females’ PAs differently, in that the odds of males engaging in active living increased in the ages of 20~60 years, but it decreased in females. Similar results in a previous study also found a trend of regular PA patterns increasing in men aged 30~64 years, but decreasing in women aged 18~64 years [42]. In future studies, interactive effects of demographic variables for PA should be examined. For example, PA between higher-income women and lower-income men could be compared.

This study also showed that individuals with higher educational levels had higher odds of participating in active living than did those with lower educational levels. Thus, educational level affects individuals’ healthy behaviors. The number of years of schooling an individual completes has a positive association with the days of vigorous PA per week [43]. While income was not a significant predictor of PA in this study, individuals of a higher socioeconomic status are more likely to participate in active living [44]. One of the possible reasons is that 40% of participants in this study were students and unemployed, and thus did not have a fixed salary, so that their income was comparatively low. Students and unemployed participants are more likely to use active transport, such as cycling, walking, or public transportation. They also have more opportunities to participate in recreation-related PAs. Public health practitioners and policymakers should pay attention to vulnerable populations who present an inactive lifestyle, such as women, older adults, and those with a low educational level. Psychological and environmental approaches can be used to encourage PA in vulnerable populations. For example, social support from significant others and engaging in PA together with family, friends, and neighbors can encourage older adults’ PA [39].

These results suggest that individuals who are obese had a lower OR of participating in active living than individuals with a normal BMI, while people with a high BMI demonstrated a low level of PA and were more likely to engage in sedentary behaviors [45]. This study also found that individuals with cardiometabolic diseases had lower odds of participating in active living than healthy individuals, and individuals with one or more chronic diseases had a lower willingness to participate in PA than did those without a chronic condition. The number of chronic diseases reported was negatively associated with the respondent’s level of PA [46]. Insufficient PA is both a cause and a consequence of increased body weight and risks of chronic diseases [47]. Public health practitioners should encourage individuals at high risk of chronic diseases to acknowledge the perceived benefits of PA and threats to their current health condition, from a psychological perspective. Because individuals with chronic diseases more frequently go to outpatient clinics, policymakers can consider the accessibility of healthcare institutions to encourage walking behaviors.

There are several limitations of this study. First, this study employed survey methods with a convenience sampling process that involved recruiting participants through the internet. The representativeness of our sample could thus be biased. Stratified random sampling would be helpful in avoiding this bias in future studies; moreover, a better method of data collection would be to combine phone-based, interview-based, and internet-based surveys to approach a broader population. Second, the design of the IPAQ asks about PA in the previous 7 days. However, it is possible that participants answered with a retrospective view of their mean activity time in a typical week. This could have affected the results. Third, interactions among two or more demographic variables were not discussed in the current study. For example, younger men might be more physically active than older women. A cluster analysis could be used to classify participants’ patterns in future studies. In addition, there are other demographic variables that could impact PA, such as the employment status, marital status, and unhealthy behaviors. The completeness of indicators was also limited, as even the indicators of the policy environment domain were based on the previous literature. Fourth, as the HBMPA is only one of many possible explanatory theories of PA from a psychological perspective, a wider range of theoretical foundations of PA could be used in future studies. Finally, the predictive power of PA was 23.1%. There are still other predictors of PA that could be taken into consideration in future studies.

## 5. Conclusions

This study provides support for the Ecological Model of Active Living. The environmental determinants of PA include demographic variables, the walkability index, perceived accessibility and walkability, and the HBMPA. In order to achieve the goal of active living and sufficient PA requirements, cooperation of individuals, significant others, the environment, and the government is necessary. Health education and physical education in schools, in the workplace, and in the community could elevate an individual’s attitudes toward PA. Before policy implementation, the walkability of the environment and the accessibility of infrastructure should be taken into consideration in policy planning for the perceived environment and behavior setting domains. The predictive path model is a good approach to quantitatively measure impacts on active living of various determinants that suggests further lines of research in approaches for modeling these relationships.

## Supporting information

S1 File Data. (XLSX)
