# Correction: Determinants of physical activity: A path model based on an ecological model of active living

**DOI:** 10.1371/journal.pone.0222625

**Published:** 2019-09-12

**Authors:** Hsin-Yen Yen, Ching Li

The first author, Hsin-Yen Yen, is incorrectly labeled as the corresponding author. The correct corresponding author is Ching Li. Dr. Li’s email address is: t94002@ntnu.edu.tw.
